# Single Cell Analysis in Vascular Biology

**DOI:** 10.3389/fcvm.2020.00042

**Published:** 2020-03-31

**Authors:** Nicholas W. Chavkin, Karen K. Hirschi

**Affiliations:** ^1^Department of Cell Biology, Developmental Genomics Center, School of Medicine, University of Virginia, Charlottesville, VA, United States; ^2^Departments of Medicine and Genetics, Cardiovascular Research Center, School of Medicine, Yale University, New Haven, CT, United States

**Keywords:** vascular development, genomic analysis, vascular disease, single cell analysis, bioinformatics

## Abstract

The ability to quantify DNA, RNA, and protein variations at the single cell level has revolutionized our understanding of cellular heterogeneity within tissues. Via such analyses, individual cells within populations previously thought to be homogeneous can now be delineated into specific subpopulations expressing unique sets of genes, enabling specialized functions. In vascular biology, studies using single cell RNA sequencing have revealed extensive heterogeneity among endothelial and mural cells even within the same vessel, key intermediate cell types that arise during blood and lymphatic vessel development, and cell-type specific responses to disease. Thus, emerging new single cell analysis techniques are enabling vascular biologists to elucidate mechanisms of vascular development, homeostasis, and disease that were previously not possible. In this review, we will provide an overview of single cell analysis methods and highlight recent advances in vascular biology made possible through single cell RNA sequencing.

## Introduction

Recent advancements in single cell analyses have enabled researchers to investigate tissue development, cell heterogeneity, and cellular response to injury and disease in unprecedented ways. Such analyses require continued development of techniques that separate and label single cells isolated from tissues, quantify components in each cell (RNA, DNA, protein, etc.), and analyze resulting data on a single cell level. These bioinformatic pipelines can cluster similar cells, compare gene expression patterns among clusters, and predict their signaling and lineage progression pathways. Comparing individual cells from a tissue-wide perspective has revealed previously unappreciated levels of cellular heterogeneity, identified unknown cell types and elucidated cellular differentiation and interaction pathways.

**Figure 1:**
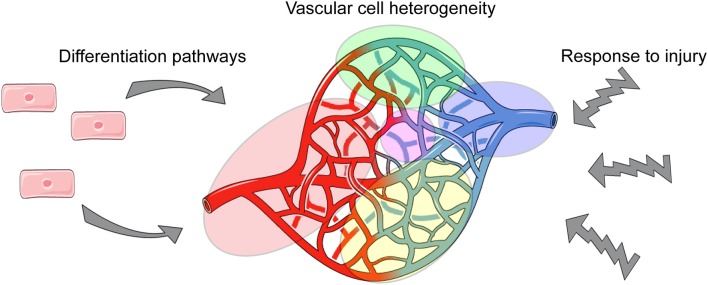
Applications of single cell analysis to understanding aspects of vascular biology.

Obtaining such novel insights will be particularly useful for the vascular biology field, as vascular cells are abundantly distributed throughout all tissues and must establish and maintain different functions to ensure tissue homeostasis. Thus, via single cell analyses, we can define tissue-specific phenotypes and functions of vascular cells, and provide needed insight into how they cross-talk with tissue-specific cells (Figure 1).

To date, the most abundantly used single cell analysis technique has been single cell RNA sequencing (scRNA-seq) that involves generating barcoded converted DNA from RNA of single cells, transcriptome sequencing, and raw data processing. However, emerging techniques in single cell analysis that are focused on chromatin availability and protein abundance have recently allowed for exciting experiments that will deepen our understanding of vascular cell heterogeneity beyond RNA expression. In this review, we provide an overview of the scRNA-seq process, discuss the unique advantages of single cell analysis that have been used in vascular biology, and highlight emerging new single cell techniques.

## Overview of Single Cell RNA Sequencing

**Figure 2:**
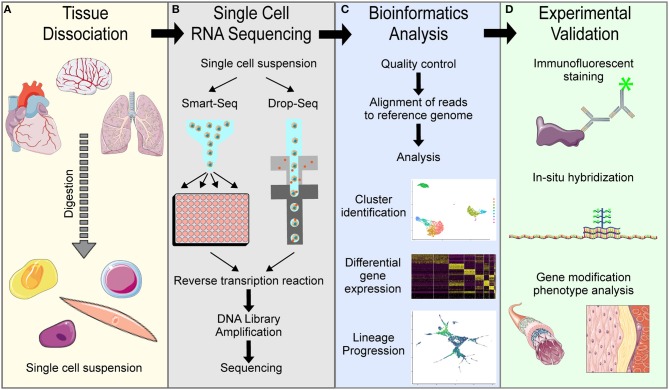
Overview of general single cell RNA sequencing pipelines. **(A)** Tissue dissociation is achieved using digestion enzymes to generate a single cell suspension. **(B)** Single cell RNA sequencing of the single cell suspension in either Smart-Seq or Drop-Seq methods to obtain single cell transcriptomes. **(C)** Bioinformatics analysis of the raw sequencing data is needed to interpret results. **(D)** Experimental validation is necessary to confirm computational findings.

In the vascular biology field, single cell RNA transcriptome sequencing has become standard for understanding cellular heterogeneity and lineage progression. The first description of whole transcriptome sequencing on the single cell level was published in 2010 by Tang et al., who dissociated cells from tissues, manually selected single cells to isolate RNA and amplify cDNA to sequence the entire transcriptome of each single cell, analyzed the data using basic bioinformatics approaches, and experimentally validated the single cell data (1). Since then, technical advances have updated some aspects of this process, but the general scRNA-seq pipeline remains the same (Figure 2): tissue dissociation, single cell barcoding and RNA sequencing, bioinformatics analysis and experimental validation.

### Single Cell Isolation

The very first step toward single cell analysis is enzymatic digestion of tissues into single cell suspensions. Isolation of healthy single cells from tissues is required to obtain high quality, reproducible data, and optimizing digestion of specific tissues is critical (2, 3). Over-digestion can induce cell death, and under-digestion can exclude subpopulations of cells from the analysis (4). Cell damage associated with enzymatic digestion can also induce changes in gene expression in specific populations of cells within tissues (5). Therefore, a digestion protocol that optimizes enzyme concentration and time of digestion is needed to isolate cell types with minimal induction of apoptosis and aberrant gene expression. Recent publications have described digestion methods for specific tissues, including heart (6), brain (7), lung (8), kidney (9, 10), and cultured cells (11, 12), which have distinct extracellular matrix densities that require different enzymes and digestion times. Digestion cocktails can include enzymes such as trypsin, collagenase and elastase, or build on predesigned cocktails like Liberase (Sigma). Recent advances in digestion techniques also include cold-activated protease methods that better preserve RNA expression patterns compared to traditional digestion methods (2). Optimizing this process to obtain a healthy suspension of single cells from the desired tissue leads to more reliable data.

### Single Cell RNA Sequencing

Delineating the transcriptome of single cells from a population requires a method for identifying sequencing reads generated from each cell. Two methods have emerged as the most-used by researchers: plate-based sequencing (Smart-seq) and droplet-based sequencing (Drop-seq). Smart-seq, as well as more recently developed Smart-seq2 (13), is a plate-based single cell isolation technique that employs flow cytometry to sort single cells into individual wells of a tissue culture plate. In each well, RNA is lysed, converted to cDNA by reverse transcriptase with barcoded primers to distinctly label reads from each cell, and then the DNA is amplified to allow for next-generation sequencing (14). Plate-based sequencing methods are commercially available through products like the Fluidigm C1 platform (Fluidigm, San Francisco, CA, USA). Additionally, variations on plate-based sequencing have been developed, such as Cell Expression by Linear Amplification and Sequencing [CEL-seq (15) and CEL-seq2 (16)], which combines the barcoded cDNA libraries and amplifies them together. The other method for scRNA-seq is droplet-based sequencing, Drop-seq. This method employs a microfluidics approach to suspend single cells within droplets containing reverse-transcriptase enzymes and barcoded primers (17). Drop-seq can be used to quickly emulsify large numbers of single cells and generate cDNA libraries, and has a commercially available pipeline through 10× Genomics machines and reagents (Chromium, 10X Genomics, Pleasanton, CA, USA). Comparing the scRNA-seq methods, plate-based single cell sequencing has more coverage of the transcriptome and can identify more genes per cell; whereas, Drop-seq has less amplification noise and can analyze more cells per sample with a more cost-effective approach (18). Since each method has strengths and limitations, the choice of scRNA-seq method is determined by the experimental design and research goals. The sequencing efficiency and depth of genomic analysis will vary based on the reagents used and the quality/experience of the sequencing facility.

### Computation Analysis of Single Cell RNA Sequencing Data

Understanding the data generated from scRNA-seq requires bioinformatics approaches. Many different computational-based methods have been generated for different types of analyses of raw scRNA-seq datasets. Before transcriptome analysis, it would be useful to determine whether the raw sequencing data are of sufficient quality with regard to the raw read output (per base content, Phred scores, GC and N content, sequence duplication, adapter content, etc.). Thus, using programs such as FastQC (Babraham Bioinformatics) could prevent loss of time and resources on extensive analyses of low-quality sequencing data. Additionally, sequence reads per cell and genomic coverage should be sufficient for each experiment, which can be checked in scRNA-seq analysis programs CellRanger (10× Genomics) and Seurat v3 (19) (explained below). Once high-quality sequence data has been verified, the first step in analysis is to align the reads to a reference transcriptome to convert the raw sequencing data to transcript counts. This can be done with more classic bulk-RNA sequencing approaches, including Bowtie2 (20), BWA (21), and Kallisto (22). Subsequently, the data must be de-multiplexed to assign transcript reads to individual cells.

Similarities and differences among cells can be visualized and quantified using different methods depending on the desired outcomes. The Seurat v3 program can be used to group cell types into clusters and identify key genes that are significantly different among the clusters (19), and programs such as the Monocle2 can be used to identify potential lineage connections among cell types (23, 24). Other well-defined analysis programs include BackSPIN (25) and TSCAN (26). Additionally, 10× Genomics reagents allow for straight-forward conversion from sequencing data to aligned reads and generated transcript counts using their CellRanger analysis tool. Finally, it is also important to be aware of batch effects. That is, when comparing datasets from different experiments, large variations in the data may be explained by technical differences rather than biological differences, but batch effects can be computationally corrected using software programs such as Seurat v3. Other reviews have described single cell RNA sequence analysis pipelines in more detail (27).

### Validation of Computational Results

The final step in single cell RNA sequence analysis is to validate results. Each step in the pipeline can add unintended bias in the data, so false positive or negative results must be ruled out through experimental validation. Validation experiments could include tissue immunostaining or RNA spatial patterning to verify expression results, or genetic modification to test functional relevance. Fluorescent *In Situ* Hybridization (FISH) employs probes to identify RNA strands in tissue slices, and this technique is the most direct method for validating RNA expression levels in tissues. FISH protocols have now been optimized to identify small populations or low-abundance RNA constructs (28), which is necessary for many scRNA-seq validation experiments. Validated FISH-based methods of RNA spatial analysis are also commercially available from Advanced Cell Diagnostics (Bio-Techne, Advanced Cell Diagnostics, Hayward, CA, USA) through their RNAscope products and reagents (29). RNAscope methods have also been applied to whole-mount tissues (30). Overall, experimental validation is critical for confirming single cell bioinformatics analysis.

### Online Databases of Single Cell RNA Sequencing Results

An exciting consequence of the collective genomic efforts within many fields has been the push for publicly available sequencing datasets. The Mouse Cell Atlas is a project that aims to sequence mouse tissues from as many different sources as possible, and is publishing the findings online (31). PanglaoDB is a searchable online database of single cell datasets with over 1,000 mouse and 300 human samples integrated into an easy-to-use search tool that incorporates unbiased cluster annotations (32). More specific to vascular biology, EndoDB is an online database that includes 360 datasets from bulk and scRNA-seq covering six species (33). In addition, EC Atlas is a searchable database of endothelial cell scRNA-seq data from 12 different mouse tissues (34). As more studies are performed and datasets are made public, a more complete collection of species- and tissue-specific single cell data will be searchable and usable by all researchers.

## Single Cell Advancements in Mechanisms of Vascular Development

Vascular development requires the differentiation of endothelial cells from mesodermal progenitors, and their specification toward diverse phenotypes including arterial, capillary, venous, lymphatic, and hemogenic endothelial cells (35). These developmental pathways have multiple transition states at different times during gestation. The cell signaling mechanisms driving these specification events have been somewhat defined, but *in vivo* studies have been limited by our lack of understanding of the phenotypic transitions of endothelial cells during these specification events and the low numbers of cells that can studied in developing embryonic tissues. Development of supporting mural cells in the vasculature, including pericytes and vascular smooth muscle cells (VSMC), has also been difficult to characterize. Pericytes and VSMC derive from multiple embryonic sources and have different phenotypes in adult tissues and disease (36, 37). However, scRNA-seq allows for analysis of low-abundant populations of vascular cells that exist during the transition toward mature fates, which will lead to a better understanding of vascular development.

### Primordial Endothelial Cell Characterization

Primordial endothelial cells are derived from mesodermal progenitors in the early mouse embryo at around embryonic day (E)7.5–8 (35) via a process referred to as vasculogenesis. They are then specialized to become arterial, venous and capillary endothelial cells, and primordial endothelial cells are difficult to study, *per se*, because they represent an intermediate endothelial cell state that is short-lived in the embryo and varies based on location. ScRNA-seq of mouse embryos at E8.25 revealed characteristics of primordial endothelial cells, described as branching away from mesodermal cells and containing allantois- and non-allantois-derived populations (38). Thus, delineating the transcriptome of primordial endothelial cells within developing tissues, and predicting and validating their lineage trajectory, will provide needed insights into mechanisms of vasculogenesis (39, 40). Toward this goal, stem cell culture models have also been used to recapitulate the process of endothelial cell differentiation from mesodermal progenitors and study underlying mechanisms. One such study used scRNA-seq of mouse embryonic stem cells undergoing differentiation to better characterize hemangiogenic progenitors as derived from FLK1-positive mesodermal progenitors, and identified SRC kinase as a key regulator of this transition (41). Furthermore, a recent study using scRNA-seq to analyze human pluripotent stem cell-derived hematopoietic organoids suggests that RAG1 could be a novel marker of hemogenic progenitors derived from primordial endothelial cells, as RAG1 is co-expressed with known endothelial/hematopoietic progenitor markers (CD34, VE-Cadherin and CD90) at the single cell level (42).

### Arteriovenous Endothelial Cell Specification

To form a functional blood circulatory system, primordial endothelial cells must be specified into arterial and venous fates (43). Arterial and venous blood vessels have different functions within the circulatory network, and the endothelial cells that line arteries and veins reflect those differences in gene expression, structure, and response to cytokines and growth factors (44, 45). Some of the molecular mechanisms underlying the development of mature arteries and veins have been revealed [reviewed in (43)]; however, the process is still poorly understood and further complicated by the fact that arteriovenous plexi are further phenotypically specialized to perform tissue-specific functions (46, 47), as discussed below. Nonetheless, recent studies applying scRNA-seq to heterogeneous populations of endothelial cells isolated from adult tissues, such as the cortex and lung (25, 48, 49), have revealed a surprising heterogeneity, even among endothelial cells with known arterial and venous characteristics. Thus, more work is needed to define endothelial cell subtypes throughout the body and gain insights into their differentiation pathways and molecular regulation.

Studies using human embryonic stem cells (hESC) and induced pluripotent stem cells (iPSC) can lend further insight into the process of endothelial cell specification in a controlled environment. Endothelial cell differentiation from hESC and iPSC has been described [reviewed in (50)], and iPSC-EC appear to be functionally heterogeneous (51). ScRNA-seq confirmed phenotypic differences among iPSC-EC; e.g.„ one differentiation protocol yielded four distinct endothelial subtypes (52). Differentiation protocols to promote arterial and venous specification of human stem cell-derived endothelial cells have also been established (53, 54). ScRNA-seq of such human endothelial cells suggests differentiation pathways consistent with previous *in vivo* studies including TGF/BMP signaling, Notch activation, and shear stress (43, 55, 56). Further evaluation and validation of scRNA-seq data from human stem cell-derived endothelial cells is likely to reveal new mechanisms of arteriovenous differentiation, as well.

### Endothelial-to-Hematopoietic Transition

During development, another distinct type of endothelial cells, termed hemogenic endothelial cells, are also specified and give rise to the hematopoietic stem and progenitor cells that serve as the foundation of the hematopoietic system. Hemogenic endothelial cells are known to form in the extraembryonic yolk sac and placenta, as well as within the aorta-gonad-mesonephros (AGM) region of the embryo (57). The number of hemogenic endothelial cells in each of these tissues is very small [~1–3% of endothelial cells; (35, 58, 59)], and the process of endothelial-to-hematopoietic (EHT) involves a progression through multiple intermediate cell types; thus, hemogenic endothelial cells and the EHT process are difficult to study without scRNA-seq. The same experiment that investigated primordial endothelial cells in the E8.25 embryo also found a small population of endothelial cells that had potential to generate hemogenic endothelium, which were characterized by expression of leukotriene production controlling Alox5 gene expression (38). A separate study investigated endothelial cells at E11.5 via scRNA-seq and found that the small population of hemogenic endothelial cells in the AGM exhibited enriched expression of transcription factors Elk3, Jun, and Mecom (60). Thus, the differential expression of genes in the small population of hemogenic endothelial cells compared to non-blood forming vascular endothelium will likely be better characterized using single cell analysis approaches.

### Pericyte and Vascular Smooth Muscle Cell Differentiation

Mural cells, including pericytes and VSMC, are required for blood vessel formation and maturation (61). Understanding the differentiation of these cells that make up the vessel wall is therefore critical for understanding vascular development. For example, in the brain, pericytes are critically important for the development and maintenance of the blood-brain barrier (62, 63); however, pericytes, in general, have been poorly defined and lack universal markers for identification. Thus, they have been thought to represent a heterogeneous population of cells with different functions and their differentiation pathways have not been clearly defined (64, 65). However, recent scRNA-seq of mural cells isolated from adult cortex suggest that pericytes within this tissue reside within one clearly defined population (48); thus, similar studies of pericytes derived from other tissues should provide needed insights into tissue-specific phenotypes and functions of these vascular cells.

VSMC are known to be derived from multiple embryonic lineages *in vivo* (66), which has led to difficulty in understanding their developmental pathways. To address this issue, a recent study performed scRNA-seq of the cardiac outflow tract at early-, mid-, and late-stage development. These studies revealed that although the VSMC within this tissue are derived from different sources (mesoderm and neural crest), they converge to the same phenotype [i.e., myocardial-to-VSMC and mesenchymal-to-VSMC (67)] through different developmental pathways. Further study of VSMC from many tissues is also needed to understand the diversity of their phenotypes, functions and molecular regulation. Additionally, studies using human pluripotent stem cells could provide more needed insight; e.g., one such study suggests that pericytes, stromal cells, and vascular smooth muscle cells could originate from a clonal mesenchymal progenitor cell type, referred to as mesenchymoangioblasts (PDGFRβ^+^CD271^+^CD73^−^) (68). Clearly, more work is needed in multiple complementary model systems.

## Tissue-Specific Differentiation of Vascular Cells

Vascular cell phenotypes and functions vary among tissues due to tissue-specific microenvironments and requirement for diverse vascular functions (69, 70). This need for tissue-specific function leads to tissue-specific differentiation and specialization of vascular cells, which has been highlighted by recent analyses of tissue development through scRNA-seq.

### Cardiac Vascular Differentiation

One very important endothelial specification event occurs in the developing coronary vasculature where it has been shown that endothelial cells from the sinus venosus give rise to the coronary arteries. It was previously reported that sinus venosus endothelial cells proliferate in response to VEGF-C stimulation and migrate in response to shear stress-induced activation of the CXCL12-CXCR4 pathway (71–73). However, their differentiation into arterial endothelial cells was not well-described until coronary artery development was analyzed using scRNA-seq (74). This study showed a gradual specification from a venous to pre-arterial fate, and subsequent generation of coronary arteries in response to the onset of blood flow. Additionally, this study confirmed that the venous-enriched transcription factor, NR2F2 (COUP-TFII), actively inhibits Notch-induction of arterial identity (75–77). Pre-arterial cells that give rise to coronary arteries are too few in number with no previously known markers to be found in conventional analyses. This novel mechanism of venous-to-arterial phenotype transition also provides insights needed to optimize vascular interventions that induce the same transition, including arteriovenous fistulas in hemodialysis patients (78, 79), deep vein arterialization in claudicated limb ischemia patients (80, 81) and autologous vessel grafts in coronary bypass surgery patients (82).

### Neurovascular Differentiation

Endothelial cell specialization is also critically important in the brain for the generation and maintenance of the blood-brain barrier. Vascular endothelial cells in the brain contain specialized tight junctions that are more restrictive to cell and soluble factor trafficking (83, 84). The maintenance of tight junctions in brain endothelial cells is known to be dependent on Wnt signaling (85–87); however, the signaling pathways responsible for the development of endothelial barrier function has been difficult to study *in vivo*. Performing scRNA-seq of E14.5 brain endothelial cells, and comparing these data to bulk RNA sequencing of wild-type and Beta-catenin deficient brain endothelial cells, revealed that Wnt signaling is also required for the establishment of barrier function in brain endothelial cells (88). This study also identified brain endothelial-specific transcription factors Foxf2, Foxl2, Foxq1, Lef1, Ppard, Zfp551, and Zic3, and bioinformatic analysis was then used to identify subsets of brain endothelial cells with low- and high-expression of these newly discovered transcription factors. Further analyses of such cells will continue to advance our understanding of the regulation of blood-brain barrier formation and maintenance.

### Lung Vascular Differentiation

Vascular cells in the lung are unique because the flow of oxygenated blood in the lung is opposite in arteries and veins relative to other tissues in the body. In addition, there is increased need for gas exchange in the lung vasculature. ScRNA-seq analysis of cells in the lung showed that the vascular cells are comprised mainly of blood vascular endothelial cells, a small population of lymphatic endothelial cells, and two sub-populations of pericytes (49). The subpopulations of pericytes could be divided into Acta2-high and Pdgfrb-high, and the distinct endothelial cell populations exhibited differences in transcriptional regulators. Lymphatic endothelial cells were enriched for Prox1, Hoxd8, and Maf; whereas, blood vascular endothelial cells were enriched for Epas1, Klf4, Gata2, Klf2, and Sox17 (49). Further study of these potential key drivers of lung endothelial cell differentiation will advance our understanding lung vascular development. Other single cell analyses of whole lung cells will also lend to our understanding of vascular diversity in this tissue. For example, a recent scRNA-seq study of whole lung cells revealed multiple endothelial cell types in this tissue, as well as endothelial cell crosstalk with, and regulation by, alveolar epithelial type I cells and mesenchymal cells that is conserved in multiple mammalian animals (89).

## Vascular Heterogeneity

Large-scale tissue-specific vascular gene expression profiles have been revealed through comparative next-generation RNA sequencing studies (90). A *post-hoc* analysis of published single cell studies revealed that organ-specific endothelial cells were more variable between organs than within organs (91). However, recent scRNA-seq studies have also shown that vascular cells, even within the same tissue, are broadly heterogeneous. Vascular cells can be generally defined as arterial, capillary or venous depending on their location within a vascular plexus (44). In a recent scRNA-seq study, endothelial cells from 12 different mouse tissues were analyzed and compared to identify tissue-specific gene expression. The analysis revealed a broad diversity of endothelial-expressed genes among arterial, capillary and venous populations in distinct tissues (34). There were uniquely upregulated genes and functional pathways in endothelial cells from each tissue analyzed; e.g., membrane transporters in the brain; genes that control sugar metabolism in the liver; and interferon signaling in the kidney. Consistent with previous scRNA-seq reports, this new study also suggests that endothelial cells exist on a continuum from arterial to venous phenotypes, making it difficult to identify capillary-specific endothelial cell markers. Nonetheless, by comparing endothelial cells from different tissues, genes consistently specific to arterial, capillary, and venous endothelial cells among all tissues were identified. These scRNA-seq data are summarized, along with data from other studies highlighted in this review, in Table 1. ScRNA-seq can be used to further define these cell types and highlight their similarities and differences. Additionally, scRNA-seq and analysis can be used to compare sub-populations of vascular cells between tissues to identify tissue-specific phenotypes and functions. Collectively, such studies have led to a greater appreciation and understanding of different types of endothelial and mural cells and the roles that they play in tissue-specific vascular function.

**Table 1:** Novel and previously identified markers of vascular cell identity.

	**Vascular cell identity**	**Previously identified markers**	**References**	**Vanlandewijck et al. (48)**	**Kalucka et al. (34)**
Endothelial cells	Arterial ECs	Ephrin B2	(45)	Bmx	Clusterin
		Connexin40		Gastrokine-3	Crip1
		Hey1			Fibulin-2
		Hey2			Mecom
		Neuropillin-1			Sat1
		Sox17			Sema3G
		Alk1			
	Capillary ECs	VegfR2		Mfsd2a	Rgcc
				Car4	Sgk1
					Sparc
	Venous ECs	Ephb4	(45)	Slc38a5	ApoE
		COUP-TFII			Biglycan
		Neuropillin-2			Ctla2a
		Endomucin			IL-6st
					Ptgs1
					Thymosin Beta 10
Mural cells	Arterial VSMCs	Calponin 1	(92)	Myf9	
		Acta2			
		Tagln			
		Myh11			
	Venous VSMCs			Kcnj8	
	Pericytes	NG2	(93)	Vitronectin	
		PdgfRβ		Higd1b	
		Des		S1pr3	
				Mcam	
				Ifitm1	
				Baiap3	
				EH3	

### Neurovascular Heterogeneity

Vascular cell heterogeneity in the brain contributes to the maintenance of critical neurovascular functions. Endothelial cells, vascular smooth muscle cells, and pericytes all provide oxygen and nutrients to neurons, and also support neurovascular stem cell niches (94, 95). Maintenance of proper blood perfusion requires control of vascular cell fate specification (96, 97). Dysregulated arteriovenous specification of brain endothelial cells results in cerebral cavernous malformations that disrupt normal blood distribution and can rupture causing hemorrhage (98–101). Decreased blood perfusion in the brain can impair neural function and cause neurovascular disorders (102), and complete blockage of blood and vascular failure results in stroke (103). In addition, vascular cells respond to ischemic injury, which promotes angiogenesis, vascular remodeling, and activation of the neurovascular stem cell niche (104–107). Therefore, maintenance of proper blood circulation via mature arteries and veins is critical for healthy brain function.

Since blood vessels within the brain are widely distributed and interconnected, previous understanding of vascular subpopulations in the brain was limited by an inability to physically dissect the vasculature. Previous studies enabled the identification of a limited number of pericyte and vascular smooth muscle cell markers (92, 93) (Table 1); however, recent scRNA-seq and computational analysis of brain endothelial and mural cells enabled the identification and evaluation of brain-specific arterial-, capillary-, and venous-specific gene regulation (48). This study revealed that brain endothelial cells exist on a continuum from arterial to capillary to venous phenotypes, but mural cells exhibit more distinct arterial- and venous-specific phenotypes (48). This work also revealed a difference in the phenotypic regulation of arterial vs. venous endothelial cells and vascular smooth muscle cells. Interestingly, other studies have suggested that vascular cells differ within various sections of the brain, and recent studies of mouse brain vascular cells showed endothelial-specific gene expression patterns in the cortex and hippocampus (25). Even more specialized in function is the subventricular zone of the brain, which contains a neurovascular niche that maintains neural stem cells (108, 109). The population of vascular cells within the neural stem cell niche has been difficult to study, limiting our understanding of their role in neural stem cell maintenance; however, a recent scRNA-seq study showed that endothelial cell regulation of neural stem cells in the subventricular zone is mediated via Wnt and BMP signaling pathways (110). Thus, scRNA-seq studies of the brain vasculature have helped to identify specialized subtypes and their regulatory pathways.

### Cardiac Vascular Heterogeneity

In myocardial tissue, vascular cell heterogeneity is important for maintaining cardiomyocyte function. Endothelial cells are the most abundant cell type in the heart (111), and a high blood vessel density is important to keep nutrient-rich blood feeding contracting myocytes (112, 113). ScRNA-seq of healthy murine cardiac cells identified two distinct endothelial cell clusters (114); whereas, a broader analysis of multiple cardiac scRNA-seq datasets revealed three distinct endothelial cell populations: endocardial, coronary and aortic endothelial cells (91). Interestingly, vascular smooth muscle cell and pericyte heterogeneity was found to be less complex than endothelial cell heterogeneity in the heart (114, 115), and many subsets of cardiac cells were also found to express fibroblast genes (115). Single cell analysis also revealed extensive crosstalk between cell types through paracrine signaling, with abundant crosstalk pathways evident among endothelial cells, smooth muscle cells, pericytes, fibroblasts, and macrophages (115). These studies suggest that there are novel sub-populations of vascular cells in the heart that exhibit cross-functionality that may not be observed in other tissues.

### Aorta Heterogeneity

Another vascular tissue that maintains a broad range of cell types with various functions is the aorta. Aortic vascular cells must accommodate high pulsatile flow forces, and also respond to changes in blood pressure (116), flow rate (117), and lipid levels (118, 119). Thus, endothelial cells in the aorta have very specialized functions. A recent scRNA-seq study of aortic cells identified three endothelial cell clusters with distinct gene expression patterns suggesting specialization for lipoprotein handling, angiogenesis and extracellular matrix production, and lymphatic function (120). Interestingly, aortic smooth muscle cells showed surprisingly little transcriptional variation compared to endothelial cells (120). Thus, consistent with the results in the brain and heart, healthy large blood vessels appear to have more endothelial cell heterogeneity while mural cells exhibit less transcriptional variability.

### Renal Vascular Heterogeneity

Vasculature in the kidneys is not only important for maintaining renal tissue function, but also for controlling blood pressure throughout the body. Renal vascular integrity is critical for maintaining blood filtration function, where blood passes through the glomeruli and nephrons to filter out metabolites and other compounds that are then excreted through urine [reviewed in (121)]. The vasculature in the kidneys also transports renal hormones that control blood pressure through the renin-angiotensin system [reviewed in (122)]. Recent scRNA-seq analysis of adult mouse renal endothelial cells revealed extensive heterogeneity; 24 different endothelial subtypes were characterized (123). This degree of endothelial cell heterogeneity is necessary for establishing and maintaining diverse types of blood vessels in the kidney that enable distinct functions, including the renal artery and vein, as well as the fenestrated vasculature of the glomerulus, and the vasa recta in the medulla. Learning more about the unique characteristics of each of these endothelial cell subpopulations will enable a better understanding of the regulation of their distinct structures and functions. For example, this work discovered that specific medullary endothelial cell subtypes respond to water deprivation via upregulation of hypoxia response, glycolysis and oxidative phosphorylation to preserve cell viability. As proper kidney function is critical for the function of many other organs, further investigation into these renal endothelial cell types may reveal novel regulatory mechanisms that are critical for vascular functions throughout the body.

## Understanding Population Changes in Disease

Chronic and acute injury to the vascular system can induce a broad range of responses. The observed heterogeneity of vascular cells suggests that these responses may be specific to sub-types of vascular cells. Additionally, vascular cells may differentiate into disease-specific phenotypes that have new functions. Advances in scRNA-seq have allowed for the study of the differential effects of injury and disease on subpopulations of vascular cells.

### Neurovascular Disease

Maintenance of vascular integrity in the brain is critical for cognitive function. Hypoxic conditions in the brain induce rapid vascular responses to increase blood flow, including angiogenesis to form new blood vessels (106). Specialized endothelial tip cells are the leading cells in an angiogenic sprout and control the rate of angiogenesis (124). These endothelial tip cells are rare, yet one single cell analysis study of the mouse brain identified a subset of neurovascular endothelial cells that activate tip cell genes during chronic tissue hypoxia (125). This finding suggests that, in a specific population of endothelial cells in the brain, hypoxia induces a tip cell phenotype to promote angiogenesis and reperfusion. It would be interesting to apply similar approaches to other ischemic-injured tissues to determine whether such endothelial cells are ubiquitously distributed throughout the body to facilitate tissue repair.

### Responses to Myocardial Infarction

Another tissue in which ischemia induces angiogenesis is the heart. The cardiac vascular cells are very important for supplying nutrients to cardiomyocytes, and lack of proper blood supply leads to heart failure (126, 127). Myocardial infarction is the most dramatic form of myocardium tissue ischemia and results in a large amount of cell death and subsequent angiogenesis (128–130). Single cell analysis of hearts after myocardial infarction shows separation of arterial, capillary and venous endothelial cell clusters, and identifies strong interactions between vascular endothelial cells and fibroblasts (131). Additionally, following myocardial infarction, endothelial cells within the heart were analyzed using scRNA-seq, which identified Plasmalemma vesicle-associated protein (Plvap) as a novel gene upregulated specifically in a subset of cardiac endothelial cells that clonally expand near the fibrotic scar (132). In subsequent validation studies, Plvap was confirmed to regulate endothelial cell proliferation, suggesting a pro-angiogenic role in this endothelial subset during neovascularization following myocardial infarction. This study also showed the upregulation of some genes involved in endothelial-to-mesenchymal transition following myocardial infarction; however, this upregulation was not accompanied by downregulation of endothelial genes leading the authors to conclude that endothelial-to-mesenchymal transition did not play an obvious role in post-infarction neovascularization. Together, these studies show that, just as in the brain, different types of endothelial cells respond differently to cardiac injury, and suggest that cardiac endothelial cell heterogeneity is important for managing the response to injury.

### Arteriovenous Specification in Disease

Specification and maintenance of arterial and venous endothelial cells is critical for tissue survival and function; however, dysregulation of arteriovenous specification can occur spontaneously or can be induced. For example, an arteriovenous fistula (AVF) occurs when an artery and vein are shunted so that blood flow avoids a capillary network. AVF are usually created surgically to improve access for hemodialysis patients with end-stage renal disease (133). This causes large increases in shear flow force in the venous blood vessel, which requires remodeling of both endothelial and mural cells to strengthen the venous structure and enable arterial function (134, 135). But the surgical AVF can become stenotic and fail. Interestingly, single cell isolation and sequencing of endothelial cells from patients with healthy or stenotic arteriovenous fistulas showed four separate endothelial cell clusters, each containing cells from both healthy and stenotic vessels; however, stenotic vessels had differential regulation of SMAD4 (136). Arteriovenous specification is strongly linked to SMAD4 and TGF/BMP signaling, as disruption of this signaling pathway results in arteriovenous malformations during development (137, 138). This single cell analysis suggests that dysregulated arteriovenous specification via SMAD4 may contribute to failing AVF.

### Arterial Diseases

Large arteries are critical for maintaining blood pressure and flow regulation to tissues. Injury to large arteries can include damage to the endothelial cell layer and VSMC-mediated atherosclerotic plaque formation. VSMC contribute to atherosclerotic plaque formation via proliferation and trans-differentiation (139), and interestingly, VSMC contribution to plaque formation is a clonal process, typically arising from a single VSMC in the medial layer of large vessels (140). ScRNA-seq has also revealed an arterial Sca1-positive cell population that derives from the arterial adventitia and contributes to VSMC expansion in response to arterial injury (141). Additionally, endothelial cells in the lumen of large arteries respond to injury by clonal expansion (142). These cells may be tissue-resident endothelial progenitor cells with greater regenerative properties (143), suggesting that endothelial cell heterogeneity can contribute to vessel repair after injury (144).

Vascular endothelial and smooth muscle cell heterogeneity is also apparent within arterioles in different tissues, which may also play a role in tissue response to injury. Maintenance of proper blood vessel diameter in arterioles requires signal transduction through G-protein coupled receptors (GPCR), but there are many different GPRC with varying functions and downstream signaling targets (145). Single cell analysis showed that expression of these GPCR is highly variable within endothelial and smooth muscle cells of arterioles within different tissues, but also among vascular cells of the same tissue (146). Patterns of GPCR expression unique to endothelial cells in the lungs (Glp1r, Ccbp2, Lphn3, Celsr2), skeletal muscle (Ednrb, Ptger4, Adora2a, F2r), and brain (Gpr4, Gpr124, Gpr30, Lpar4) were identified. Similarly, in VSMC, the expression of some GPCR was specific to aorta (e.g., Lphn2, Cmklr1, Lpar1) and skeletal muscle (e.g., Ednrb, Adora2, Gpr30). This observed cell type and tissue-specific variability in GPCR expression suggests that arterioles may respond to injury through different signaling pathways. Additionally, the anti-inflammatory and anti-proliferative GPCRs Ptgir and Vipr2 were found to be upregulated specifically in dedifferentiated VSMC in atherosclerotic plaques suggesting they may play a role in clonal vascular cell response to injury.

### Tumor Vasculature

Vasculature in tumors is structurally and functionally different from vasculature in healthy tissues. Endothelial cells within tumor blood vessels have increased VEGF and Dll4-Notch signaling, which leads to increased proliferation and permeability, enabling leakage and metastasis (147–149). The consequences of treating tumors in mice with anti-VEGF or anti-Dll4 treatments was analyzed by scRNA-seq to find that the number of tip cells, a marker of angiogenesis, was reduced in anti-VEGF, but not anti-Dll4, treatment (150). These results suggest that initiation of angiogenesis via tip cell activation in tumor vasculature is specifically mediated through VEGF signaling. This novel understanding of tumor vascular regulation can help optimize anti-cancer treatments that target the dysregulated vasculature for drug delivery or attempt to mature the tumor vasculature to inhibit metastasis (151).

### Age-Related Macular Degeneration

Occurring in 8.7% of people aged 45–85, Age-Related Macular Degeneration (AMD) is one of the most prevalent disorders that leads to blindness (152). In AMD, damage to the retina progresses gradually and results in loss of vision and eventual blindness (153). The most common treatment for AMD is anti-VEGF therapy, suggesting neovascularization may play a role in disease progression (154–156); however, the underlying cause of AMD is not well-defined. A recent scRNA-seq study evaluated human AMD tissues and found that, among all vascular cells, one specific cell type exhibited functional changes associated with AMD progression, along with glia and cone photoreceptor cells (157). Further single cell studies of the functional changes in specific vascular cell types responsible for AMD progression could lead to improved therapies that target putative driver cell types.

## Future Applications of Single Cell Analysis in Vascular Biology

Novel single cell analysis techniques are being continually generated. Advances in sequencing and computational analyses are helping to improve scRNA-seq, but new approaches are not limited to evaluating RNA expression. Chromatin accessibility and high-dimensionality protein assays are also being optimized for single cell analysis. All of these techniques can be combined to understand cell heterogeneity on multiple molecular levels.

### New Single Cell RNA Sequencing Methods

New advances in scRNA-seq techniques include the development of Quartz-Seq2, a new method that allows for 30–50% more gene reads per cell compared to established methods (158). Novel advanced computational analysis techniques are also being developed to address limitations of scRNA-seq. One limitation, the abundance of technical noise that can lead to reduced gene reads, is being addressed with a new computational technique termed Markov affinity-based graph imputation of cells (MAGIC), which uses statistical approaches to recapitulate the transcript values lost from technical errors and dropout (159). An additional limitation to scRNA-seq is the lack of spatial recognition of scRNA-seq. A novel imaging-based approach attempts to solve this limitation. The fluorescent *in situ* RNA sequencing (FISSEQ) method starts with fixed cells and tissues, converts RNA to cDNA, and uses sequencing reagents and sensitive confocal microscopy to manually read the sequence (160, 161). In this manner, the spatial aspect of transcript heterogeneity is preserved.

### Chromatin Accessibility With Single Cell ATAC Sequencing

Beyond scRNA-seq, advancements in other types of single cell analyses are emerging. For example Assay for Transposase-Accessible Chromatin (ATAC) sequencing can determine regions of open chromatin by using transposase to insert specific primers to sequence open regions of DNA (162). This technique has been optimized for low cell numbers (163), and has recently been applied to single cells. Single cell ATAC sequencing has been described with a microfluidics platform (163), and a sorting-based method to isolate single cells (164). A bioinformatics approach called Destin has been designed specifically to analyze single cell ATAC sequence reads (165). More advanced techniques and standardized protocols will help the vascular biology field define cell heterogeneity on the DNA epigenetic level.

### Protein Heterogeneity With CyTOF and Cell Signaling

Single cell protein analysis has also had some exciting recent advances. Traditional single cell approaches to protein analysis have used flow cytometry to define populations of cells, but the degree of variation described in flow cytometry experiments is limited by availability of excitation and emission ranges. However, a new method of flow cytometry, Mass cytometry time-of-flight (CyTOF), uses metallic-bound antibodies to read the metal ions attached to cells through the antibody interactions, allowing for many more antibodies to be used in a single experiment (166). This method adds more variables to better describe and understand cell heterogeneity on a protein level. However, CyTOF still requires dissociation of cells. A new method called multiplexed ion beam imaging (MIBI) applies the metal ion-based approaches of CyTOF to fixed tissue slices, allowing for spatial resolution of protein heterogeneity (167). Together, these approaches will help define protein heterogeneity among vascular cell types.

## Discussion

Single cell analysis has revolutionized our understanding of vascular biology. Vascular cell differentiation, functional heterogeneity, and population-specific response to disease have all been characterized in greater detail than previously possible. Such studies have also suggested trends among vascular cells; e.g., endothelial cells appear to have a high degree of heterogeneity, while mural cells exhibit less phenotypic diversity. Additionally, we have gained insights into tissue-specific differentiation of vascular cells that plays a key role in establishing diverse functions including blood-brain barrier, cardiac vascular density and aortic vascular cell responses.

The process of vascular cell differentiation (or dedifferentiation) and specialization in development and disease represents a continuum of fate changes, and single cell analysis is particularly well-suited for identifying transitional phenotypes along a trajectory. Additionally, the novel hypothesis that vascular cells in an established plexus may exist in a continuum of arteriovenous identity can be more rigorously tested using advanced single cell analysis (25). Finally, single cell analysis will provide needed insight into clonal regenerative potential of specific vascular cell subtypes in response to injury or disease (144). Thus, future studies using different types of single cell approaches will enable a more in-depth investigation of the establishment of vascular cell types and regulation of their functions. Although most current studies are focused on transcriptional analysis, gaining further understanding of the role of epigenetic and protein regulation in vascular cell differentiation, tissue-specific function, and disease response is also needed. Thus, continued single cell analysis studies of all types will lead to a better understanding of the diverse phenotypes and functions of vascular cells.

Advances in single cell analysis will ultimately improve our ability to maintain health and treat disease. Understanding cell heterogeneity and differential cell population-specific response to disease has large implications for vascular therapies. Targeted medicine approaches can take advantage of vascular cell heterogeneity to manipulate precise cell types for drug delivery or gene therapy (168–170). For example, drug delivery via homing peptides could potentially target tissue-specific vasculature (171) or tumor vasculature (172). Overall, future therapeutic strategies will need to understand vascular cell heterogeneity on multiple levels to generate targeted, specific therapies for prevalent diseases.

## Author Contributions

All authors listed have made a substantial, direct and intellectual contribution to the work, and approved it for publication.

### Conflict of Interest

The authors declare that the research was conducted in the absence of any commercial or financial relationships that could be construed as a potential conflict of interest.

## References

[1] Tang F Barbacioru C Bao S Lee C Nordman E Wang X. Tracing the derivation of embryonic stem cells from the inner cell mass by single-cell RNA-Seq analysis. Cell Stem Cell. (2010) 6:468–78. 10.1016/j.stem.2010.03.015 20452321 PMC2954317

[2] Potter AS Potter S. S. Dissociation of tissues for single-cell analysis. Methods Mol Biol. (2019) 1926:55–62. 10.1007/978-1-4939-9021-4_5 30742262

[3] Braga FAV Miragaia RJ. Tissue handling and dissociation for single-cell RNA-Seq. Methods Mol Biol. (2019) 1979:9–21. 10.1007/978-1-4939-9240-9_2 31028629

[4] Skulska K Wegrzyn AS Chelmonska-Soyta A Chodaczek G. Impact of tissue enzymatic digestion on analysis of immune cells in mouse reproductive mucosa with a focus on gammadelta T cells. J Immunol Methods. (2019) 474:112665. 10.1016/j.jim.2019.112665 31525366

[5] van den Brink DC Sage F Vertesy A Spanjaard B Peterson-Marudo J Baron CS. Single-cell sequencing reveals dissociatin-induced gene expression in tissue subpopulations. Nature Methods. (2017) 14:935–6. 10.1038/nmeth.4437 28960196

[6] Lee LL Khakoo AY Chintalgattu V Isolation and purification of murine cardiac pericytes. J Vis Exp. (2019) 16:150 10.3791/59571 31475977

[7] Volovitz I Shapira N Ezer H Gafni A Lustgarten M Alter T. A non-aggressive, highly efficient, enzymatic method for dissociation of human brain-tumors and brain-tissues to viable single-cells. BMC Neurosci. (2016) 17:30. 10.1186/s12868-016-0262-y 27251756 PMC4888249

[8] Happle C Meter-Decking L Dreier A Wetzke M Glasener S Grychtol R. Improved protocol for simulatneous analysis of leukocyte subsets and epithelial cells from murine and human lung. Exp Lung Res. (2018) 44:127–36. 10.1080/01902148.2018.1432721 29677457

[9] Manolopoulou M Matlock BK Nlandu-Khodo S Simmons AJ Lau KS Phillips-Mignemi M. Gewin. Novel kidney dissociation protocol and image-based flow cytometry facilitate improved analysis of injured proximal tubules. Am J Physiol Renal Physiol. (2019) 316:F847–55. 10.1152/ajprenal.00354.2018 30759021 PMC6580245

[10] Schaffer S Maul-Pavicic A Voll RE Chevalier N. Optimized isolation of renal plasma cells for flow cytometric analysis. J Immunol Methods. (2019) 474:112628. 10.1016/j.jim.2019.06.019 31254500

[11] Sekiguchi R Hauser B. Preparation of cells from embryonic organs for single-cell RNA sequencing. Curr Protoc Cell Biol. (2019) 83:e86. 10.1002/cpcb.86 30957983 PMC6506382

[12] Ikeda T Ichikawa K Shigeto H Ishida T Hirota R Funabashi H. Arginine-mediated dissociation of single cells and cell sheets from a polystyrene culture dish. Biosci Biotechnol Biochem. (2019) 83:2272–5. 10.1080/09168451.2019.1659716 31482750

[13] Picelli S Faridani OR Bjorklund AK Winberg G Sagasser S Sandberg R. Full-length RNA-seq from single cells using Smart-seq2. Nat Protoc. (2014) 9:171–81. 10.1038/nprot.2014.006 24385147

[14] Ramskold D Luo S Wang YC Li R Deng Q Faridani OR. Full-length mRNA-Seq from single-cell levels of RNA and individual circulating tumor cells. Nat Biotechnol. (2012) 30:777–82. 10.1038/nbt.2282 22820318 PMC3467340

[15] Hashimshony T Wagner F Sher N Yanai I. CEL-Seq: single-cell RNA-Seq by multiplexed linear amplification. Cell Rep. (2012) 2:666–73. 10.1016/j.celrep.2012.08.003 22939981

[16] Hashimshony T Senderovich N Avital G Klochendler A de Leeuw Y Anavy L. CEL-Seq2: sensitive highly-multiplexed single-cell RNA-Seq. Genome Biol. (2016) 17:77. 10.1186/s13059-016-0938-8 27121950 PMC4848782

[17] Macosko EZ Basu A Satija R Nemesh J Shekhar K Goldman M. Highly parallel genome-wide expression profiling of individual cells using nanoliter droplets. Cell. (2015) 161:1202–14. 10.1016/j.cell.2015.05.002 26000488 PMC4481139

[18] Ziegenhain C Vieth B Parekh S Reinius B Guillaumet-Adkins A Smets M. Comparative Analysis of Single-Cell RNA Sequencing Methods. Mol Cell. (2017) 65:631–643.e4. 10.1016/j.molcel.2017.01.023 28212749

[19] Stuart T Butler A Hoffman P Hafemeister C Papalexi E Mauck WM. Comprehensive Integration of Single-Cell Data. Cell. (2019) 177:1888–902 e21. 10.1016/j.cell.2019.05.031 31178118 PMC6687398

[20] Langmead B Salzberg LS. Fast gapped-read alignment with Bowtie 2. Nat Methods. (2012) 9:357–9. 10.1038/nmeth.1923 22388286 PMC3322381

[21] Li H Durbin R. Fast and accurate short read alignment with Burrows-Wheeler transform. Bioinformatics. (2009) 25:1754–60. 10.1093/bioinformatics/btp324 19451168 PMC2705234

[22] Bray NL Pimentel H Melsted P Pachter L. Near-optimal probabilistic RNA-seq quantification. Nat Biotechnol. (2016) 34:525–7. 10.1038/nbt.3519 27043002

[23] Trapnell C Cacchiarelli D Grimsby J Pokharel P Li S Morse M. The dynamics and regulators of cell fate decisions are revealed by pseudotemporal ordering of single cells. Nat Biotechnol. (2014) 32:381–6. 10.1038/nbt.2859 24658644 PMC4122333

[24] Qiu X Hill A Packer J Lin D Ma YA Trapnell C. Single-cell mRNA quantification and differential analysis with Census. Nat Methods. (2017) 14:309–15. 10.1038/nmeth.4150 28114287 PMC5330805

[25] Zeisel A Munoz-Manchado AB Codeluppi S Lonnerberg P Manno GL Jureus A. Cell types in the mouse cortex and hippocampus revealed by single-cell RNA-seq. Science. (2015) 347:1138–42. 10.1126/science.aaa1934 25700174

[26] Ji Z Ji H. TSCAN: Pseudo-time reconstruction and evaluation in single-cell RNA-seq analysis. Nucleic Acids Res. (2016) 44:e117. 10.1093/nar/gkw430 27179027 PMC4994863

[27] Li G Dzilic E Flores N Shieh A Wu MS. Strategies for the acquisition of transcriptional and epigenetic information in single cells. J Thorac Dis. (2017) 9(Suppl. 1):S9–16. 10.21037/jtd.2016.08.17 28446964 PMC5383559

[28] Moffitt JR Hao J Wang G Chen KH Babcock HP Zhuang X. High-throughput single-cell gene-expression profiling with multiplexed error-robust fluorescence in situ hybridization. Proc Natl Acad Sci USA. (2016) 113:11046–51. 10.1073/pnas.1612826113 27625426 PMC5047202

[29] Wang F Flanagan J Su N Wang LC Bui S Nielson A. RNAscope: a novel *in situ* RNA analysis platform for formalin-fixed, paraffin-embedded tissues. J Mol Diagn. (2012) 14:22–9. 10.1016/j.jmoldx.2011.08.002 22166544 PMC3338343

[30] Kersigo J Pan N Lederman JD Chatterjee S Abel T Pavlinkova G. A RNAscope whole mount approach that can be combined with immunofluorescence to quantify differential distribution of mRNA. Cell Tissue Res. (2018) 374:251–62. 10.1007/s00441-018-2864-4 29974252 PMC6878655

[31] Han X Wang R Zhou Y Fei L Sun H Lai S. Mapping the mouse cell atlas by microwell-Seq. Cell. (2018) 172:1091–07.e17. 10.1016/j.cell.2018.02.001 29474909

[32] Franzen O Gan LM Bjorkegren LJM. PanglaoDB: a web server for exploration of mouse and human single-cell RNA sequencing data. Database. (2019) 2019:baz046. 10.1093/database/baz046 30951143 PMC6450036

[33] Khan S Taverna F Rohlenova K Treps L Geldhof V de Rooij L. EndoDB: a database of endothelial cell transcriptomics data. Nucleic Acids Res. (2019) 47:D736–44. 10.1093/nar/gky997 30357379 PMC6324065

[34] Kalucka J de Rooij L Goveia J Rohlenova K Dumas SJ Meta E. Single-cell transcriptome atlas of murine endothelial cells. Cell. (2020) 180:764–79.e20. 10.1016/j.cell.2020.01.015 32059779

[35] Marcelo KL Goldie LC Hirschi KK. Regulation of endothelial cell differentiation and specification. Circ Res. (2013) 112:1272–87. 10.1161/CIRCRESAHA.113.300506 23620236 PMC3768127

[36] Yamazaki T Mukouyama SY. Tissue specific origin, development, and pathological perspectives of pericytes. Front Cardiovasc Med. (2018) 5:78. 10.3389/fcvm.2018.00078 29998128 PMC6030356

[37] Wang G Jacquet L Karamariti E Xu Q. Origin and differentiation of vascular smooth muscle cells. J Physiol. (2015) 593:3013–30. 10.1113/JP270033 25952975 PMC4532522

[38] Ibarra-Soria X Jawaid W Pijuan-Sala B Ladopoulos V Scialdone A Jörg DJ. Defining murine organogenesis at single-cell resolution reveals a role for the leukotriene pathway in regulating blood progenitor formation. Nat Cell Biol. (2018) 20:127–34. 10.1038/s41556-017-0013-z 29311656 PMC5787369

[39] Belaoussoff M Farrington SM Baron HM. Hematopoietic induction and respecification of A-P identity by visceral endoderm signaling in the mouse embryo. Development. (1998) 125:5009–18. 9811585 10.1242/dev.125.24.5009

[40] Vokes SA Krieg AP. Endoderm is required for vascular endothelial tube formation, but not for angioblast specification. Development. (2002) 129:775–85. 11830576 10.1242/dev.129.3.775

[41] Zhao H Choi K. Single cell transcriptome dynamics from pluripotency to FLK1(+) mesoderm. Development. (2019) 146:dev182097. 10.1242/dev.182097 31740535 PMC6918769

[42] Motazedian A Bruveris FF Kumar SV Schiesser JV Chen T Ng ES. Multipotent RAG1+ progenitors emerge directly from haemogenic endothelium in human pluripotent stem cell-derived haematopoietic organoids. Nat Cell Biol. (2020) 22:60–73. 10.1038/s41556-019-0445-8 31907413

[43] Fang J Hirschi K. Molecular regulation of arteriovenous endothelial cell specification. F1000Res. (2019) 8. 10.12688/f1000research.16701.1 31448079 PMC6668045

[44] Fish JE Wythe DJ The Molecular Regulation of Arteriovenous Specification and Maintenance. Dev Dyn. (2015) 224:391–409. 10.1002/dvdy.24252 25641373

[45] dela Paz PNG D'Amore A. Arterial versus venous endothelial cells. Cell Tissue Res. (2009) 335:5–16. 10.1007/s00441-008-0706-5 18972135 PMC4105978

[46] Corada M Morini MF Dejana E. Signaling pathways in the specification of arteries and veins. Arterioscler Thromb Vasc Biol. (2014) 34:2372–7. 10.1161/ATVBAHA.114.303218 25169934

[47] Atkins GB Jain MK Hamik A. Endothelial differentiation: molecular mechanisms of specification and heterogeneity. Arterioscler Thromb Vasc Biol. (2011) 31:1476–84. 10.1161/ATVBAHA.111.228999 21677290 PMC3134408

[48] Vanlandewijck M He L Mae MA Andrae J Ando K Del Gaudio F. A molecular atlas of cell types and zonation in the brain vasculature. Nature. (2018) 554:475–80. 10.1038/nature25739 29443965

[49] Guo M Du Y Gokey JJ Ray S Bell SM Adam M. Single cell RNA analysis identifies cellular heterogeneity and adaptive responses of the lung at birth. Nat Commun. (2019) 10:37. 10.1038/s41467-018-07770-1 30604742 PMC6318311

[50] Qiu J Hirschi KK. Endothelial cell development and its application to regenerative medicine. Circ Res. (2019) 125:489–501. 10.1161/CIRCRESAHA.119.311405 31518171 PMC8109152

[51] Rufaihah AJ Huang NF Kim J Herold J Volz KS Park TS. Human induced pluripotent stem cell-derived endothelial cells exhibit functional heterogeneity. Am J Transl Res. (2013) 5:21–35. 23390563 PMC3560482

[52] Paik DT Tian L Lee J Sayed N Chen IY Rhee S. Large-scale single-cell RNA-Seq reveals molecular signatures of heterogeneous populations of human induced pluripotent stem cell-derived endothelial cells. Circ Res. (2018) 123:443–50. 10.1161/CIRCRESAHA.118.312913 29986945 PMC6202208

[53] Lanner F Sohl M Farnebo F. Functional arterial and venous fate is determined by graded VEGF signaling and notch status during embryonic stem cell differentiation. Arterios Thromb Vasc Biol. (2007) 27:487–93. 10.1161/01.ATV.0000255990.91805.6d 17185616

[54] Sriram G Tan JY Islam I Rufaihah AJ Cao T. Efficient differentiation of human embryonic stem cells to arterial and venous endothelial cells under feeder- and serum-free conditions. Stem Cell Res Ther. (2015) 6:261. 10.1186/s13287-015-0260-5 26718617 PMC4697311

[55] Larrivee B Prahst C Gordon E del Toro R Mathivet T Duarte A. ALK1 signaling inhibits angiogenesis by cooperating with the Notch pathway. Dev Cell. (2012) 22:489–500. 10.1016/j.devcel.2012.02.005 22421041 PMC4047762

[56] Fang JS Coon BG Gillis N Chen Z Qiu J Chittenden TW. Shear-induced Notch-Cx37-p27 axis arrests endothelial cell cycle to enable arterial specification. Nat Commun. (2017) 8:2149. 10.1038/s41467-017-01742-7 29247167 PMC5732288

[57] Gritz E Hirschi KK. Specification and function of hemogenic endothelium during embryogenesis. Cell Mol Life Sci. (2016) 73:1547–67. 10.1007/s00018-016-2134-0 26849156 PMC4805691

[58] Goldie LC Lucitti JL Dickinson ME Hirschi KK. Cell signaling directing the formation and function of hemogenic endothelium during murine embryogenesis. Blood. (2008) 112:3194–204. 10.1182/blood-2008-02-139055 18684862 PMC2569173

[59] Marcelo KL Sills TM Coskun S Vasavada H Sanglikar S Goldie LC. Hemogenic endothelial cell specification requires c-Kit, Notch signaling, and p27-mediated cell-cycle control. Dev Cell. (2013) 27:504–15. 10.1016/j.devcel.2013.11.004 24331925 PMC3994666

[60] Baron CS Kester L Klaus A Boisset JC Thambyrajah R Yvernogeau L. Single-cell transcriptomics reveal the dynamic of haematopoietic stem cell production in the aorta. Nat Commun. (2018) 9:2517. 10.1038/s41467-018-04893-3 29955049 PMC6023921

[61] von Tell D Armulik A Betsholtz C. Pericytes and vascular stability. Exp Cell Res. (2006) 312:623–9. 10.1016/j.yexcr.2005.10.019 16303125

[62] Reyahi A Nik AM Ghiami M Gritli-Linde A Ponten F Johansson BR. Foxf2 is required for brain pericyte differentiation and development and maintenance of the blood-brain barrier. Dev Cell. (2015) 34:19–32. 10.1016/j.devcel.2015.05.008 26120030

[63] Esen N Vejalla A Sharma R Treuttner JS Dore-Duffy P. Hypoxia-Induced Let-7d has a role in pericyte differentiation. Adv Exp Med Biol. (2016) 923:37–42. 10.1007/978-3-319-38810-6_5 27526122

[64] Armulik A Genove G Betsholtz C. Pericytes: developmental, physiological, pathological perspectives. Dev Cell. (2011) 21:193–215. 10.1016/j.devcel.2011.07.001 21839917

[65] Pfaltzgraff ER Shelton EL Galindo CL Nelms BL Hooper CW Poole SD. Embryonic domains of the aorta derived from diverse origins exhibit distinct properties that converge into a common phenotype in the adult. J Mol Cell Cardiol. (2014) 69:88–96. 10.1016/j.yjmcc.2014.01.016 24508561 PMC4034360

[66] Majesky MW. Developmental basis of vascular smooth muscle diversity. Arterioscler Thromb Vasc Biol. (2007) 27:1248–58. 10.1161/ATVBAHA.107.141069 17379839

[67] Liu X Chen W Li W Li Y Priest JR Zhou B. Single-cell RNA-Seq of the developing cardiac outflow tract reveals convergent development of the vascular smooth muscle cells. Cell Rep. (2019) 28:1346–61.e4. 10.1016/j.celrep.2019.06.092 31365875

[68] Kumar A D'Souza SS Moskvin OV Toh H Wang B Zhang J. Specification and diversification of pericytes and smooth muscle cells from mesenchymoangioblasts. Cell Rep. (2017) 19:1902–16. 10.1016/j.celrep.2017.05.019 28564607 PMC6428685

[69] Ribatti D Nico B Vacca A Roncali L Dammacco F. Endothelial cell heterogeneity and organ specificity. J Hematother Stem Cell Res. (2002) 11:81–90. 10.1089/152581602753448559 11847005

[70] Nolan DJ Ginsberg M Israely E Palikuqi B Poulos MG James D. Molecular signatures of tissue-specific microvascular endothelial cell heterogeneity in organ maintenance and regeneration. Dev Cell. (2013) 26:204–19. 10.1016/j.devcel.2013.06.017 23871589 PMC3873200

[71] Chang AH Raftrey BC D'Amato G Surya VN Poduri A Chen HI. DACH1 stimulates shear stress-guided endothelial cell migration and coronary artery growth through the CXCL12-CXCR4 signaling axis. Genes Dev. (2017) 31:1308–24. 10.1101/gad.301549.117 28779009 PMC5580653

[72] Chen HI Sharma B Akerberg BN Numi HJ Kivela R Saharinen P. The sinus venosus contributes to coronary vasculature through VEGFC-stimulated angiogenesis. Development. (2014) 141:4500–12. 10.1242/dev.113639 25377552 PMC4302936

[73] Red-Horse K Ueno H Weissman IL Krasnow AM. Coronary arteries form by developmental reprogramming of venous cells. Nature. (2010) 464:549–53. 10.1038/nature08873 20336138 PMC2924433

[74] Su T Stanley G Sinha R D'Amato G Das S Rhee S. Single-cell analysis of early progenitor cells that build coronary arteries. Nature. (2018) 559:356–62. 10.1038/s41586-018-0288-7 29973725 PMC6053322

[75] You LR Lin FJ Lee CT DeMayo FJ Tsai MJ Tsai YS. Suppression of Notch signalling by the COUP-TFII transcription factor regulates vein identity. Nature. (2005) 435:98–104. 10.1038/nature03511 15875024

[76] Korten S Brunssen C Poitz DM Grossklaus S Brux M Schnittler HJ. Impact of Hey2 and COUP-TFII on genes involved in arteriovenous differentiation in primary human arterial and venous endothelial cells. Basic Res Cardiol. (2013) 108:362. 10.1007/s00395-013-0362-0 23744056

[77] Sorensen I Adams RH Gossler A. DLL1-mediated Notch activation regulates endothelial identity in mouse fetal arteries. Blood. (2009) 113:5680–8. 10.1182/blood-2008-08-174508 19144989

[78] Wang Y Liang A Luo J Liang M Han G Mitch WE. Blocking Notch in endothelial cells prevents arteriovenous fistula failure despite CKD. J Am Soc Nephrol. (2014) 25:773–83. 10.1681/ASN.2013050490 24480830 PMC3968498

[79] Bozzetto M Ene-Iordache B Remuzzi A. Transitional flow in the venous side of patient-specific arteriovenous fistulae for hemodialysis. Ann Biomed Eng. (2016) 44:2388–401. 10.1007/s10439-015-1525-y 26698581

[80] Schreve MA Vos CG Vahl AC de Vries JP Kum S de Borst GJ. Venous arterialisation for salvage of critically ischaemic limbs: a systematic review and meta-analysis. Eur J Vasc Endovasc Surg. (2017) 53:387–402. 10.1016/j.ejvs.2016.11.007 28027892

[81] Kum S Huizing E Schreve MA Unlu C Ferraresi R Samarakoon LB. Percutaneous deep venous arterialization in patients with critical limb ischemia. J Cardiovasc Surg. (2018) 59:665–9. 10.23736/S0021-9509.18.10569-6 29786410

[82] Ge JJ Zhao ZW Zhou ZC Wu S Zhang R Pan FM. p38 MAPK inhibitor, CBS3830 limits vascular remodelling in arterialised vein grafts. Heart Lung Circ. (2013) 22:751–8. 10.1016/j.hlc.2013.02.006 23523564

[83] Hanske S Dyrna F Bechmann I Krueger M. Different segments of the cerebral vasculature reveal specific endothelial specifications, while tight junction proteins appear equally distributed. Brain Struct Funct. (2017) 222:1179–192. 10.1007/s00429-016-1267-0 27435201

[84] Page S Munsell A Al-Ahmad JA. Cerebral hypoxia/ischemia selectively disrupts tight junctions complexes in stem cell-derived human brain microvascular endothelial cells. Fluids Barriers CNS. (2016) 13:16. 10.1186/s12987-016-0042-1 27724968 PMC5057206

[85] Lengfeld JE Lutz SE Smith JR Diaconu C Scott C Kofman SB. Endothelial Wnt/beta-catenin signaling reduces immune cell infiltration in multiple sclerosis. Proc Natl Acad Sci USA. (2017) 114:E1168–77. 10.1073/pnas.1609905114 28137846 PMC5320985

[86] Benz F Wichitnaowarat V Lehmann M Germano RF Mihova D Macas J. Low wnt/beta-catenin signaling determines leaky vessels in the subfornical organ and affects water homeostasis in mice. Elife. (2019) 8:e43818. 10.7554/eLife.43818 30932814 PMC6481993

[87] Artus C Glacial F Ganeshamoorthy K Ziegler N Godet M Guilbert T. The Wnt/planar cell polarity signaling pathway contributes to the integrity of tight junctions in brain endothelial cells. J Cereb Blood Flow Metab. (2014) 34:433–40. 10.1038/jcbfm.2013.213 24346691 PMC3948118

[88] Hupe M Li MX Kneitz S Davydova D Yokota C Kele J. Gene expression profiles of brain endothelial cells during embryonic development at bulk and single-cell levels. Sci Signal. (2017) 10:eaag2476. 10.1126/scisignal.aag2476 28698213

[89] Raredon MSB Adams TS Suhail Y Schupp JC Poli S Neumark N. Single-cell connectomic analysis of adult mammalian lungs. Sci Adv. (2019) 5:eaaw3851. 10.1126/sciadv.aaw3851 31840053 PMC6892628

[90] GTex Consortium. Human genomics: the genotype-tissue expression (GTEx) pilot analysis: multitissue gene regulation in humans. Science. (2015) 348:648–60. 10.1126/science.1262110 25954001 PMC4547484

[91] Feng W Chen L Nguyen PK Wu SM Li G. Single cell analysis of endothelial cells identified organ-specific molecular signatures and heart-specific cell populations and molecular features. Front Cardiovasc Med. (2019) 6:165. 10.3389/fcvm.2019.00165 31850371 PMC6901932

[92] Gomez D Swiatlowska P Owens KG. Epigenetic control of smooth muscle cell identity and lineage memory. Arterioscler Thromb Vasc Biol. (2015) 35:2508–16. 10.1161/ATVBAHA.115.305044 26449751 PMC4662608

[93] Smyth LCD Rustenhoven J Scotter EL Schweder P Faull RLM Park TIH. Markers for human brain pericytes and smooth muscle cells. J Chem Neuroanat. (2018) 92:48–60. 10.1016/j.jchemneu.2018.06.001 29885791

[94] Licht T Keshet E. The vascular niche in adult neurogenesis. Mech Dev. (2015) 138:56–62. 10.1016/j.mod.2015.06.001 26103548

[95] Madhok DY Vitt JR Nguyen TA. Overview of neurovascular physiology. Curr Neurol Neurosci Rep. (2018) 18:99. 10.1007/s11910-018-0905-8 30353426

[96] Kitagawa M Hojo M Imayoshi I Goto M Ando M Ohtsuka T. Hes1 and Hes5 regulate vascular remodeling and arterial specification of endothelial cells in brain vascular development. Mech Dev. (2013) 130:458–66. 10.1016/j.mod.2013.07.001 23871867

[97] Goldman SA Chen Z. Perivascular instruction of cell genesis and fate in the adult brain. Nat Neurosci. (2011) 14:1382–9. 10.1038/nn.2963 22030549 PMC3655803

[98] Schulz GB Wieland E Wustehube-Lausch J Boulday G Moll I Tournier-Lasserve E. Cerebral cavernous malformation-1 protein controls DLL4-notch3 signaling between the endothelium and pericytes. Stroke. (2015) 46:1337–43. 10.1161/STROKEAHA.114.007512 25791711

[99] Shenkar R Elliott JP Diener K Gault J Hu LJ Cohrs RJ. Differential gene expression in human cerebrovascular malformations. Neurosurgery. (2003) 52:465–77; discussion 477–8. 10.1227/01.NEU.0000044131.03495.22 12535382 PMC2709524

[100] Lampugnani MG Malinverno M Dejana E Rudini N. Endothelial cell disease: emerging knowledge from cerebral cavernous malformations. Curr Opin Hematol. (2017) 24:256–64. 10.1097/MOH.0000000000000338 28212190

[101] You C Zhao K Dammann P Keyvani K Kreitschmann-Andermahr I Sure U. EphB4 forward signalling mediates angiogenesis caused by CCM3/PDCD10-ablation. J Cell Mol Med. (2017) 21:1848–58. 10.1111/jcmm.13105 28371279 PMC5571521

[102] Iadecola C. The pathobiology of vascular dementia. Neuron. (2013) 80:844–66. 10.1016/j.neuron.2013.10.008 24267647 PMC3842016

[103] Hu X De Silva TM Chen J Faraci MF. Cerebral vascular disease and neurovascular injury in ischemic stroke. Circ Res. (2017) 120:449–71. 10.1161/CIRCRESAHA.116.308427 28154097 PMC5313039

[104] Hatakeyama M Ninomiya I Kanazawa M. Angiogenesis and neuronal remodeling after ischemic stroke. Neural Regen Res. (2020) 15:16–9. 10.4103/1673-5374.264442 31535636 PMC6862417

[105] ElAli A Theriault P Rivest S. The role of pericytes in neurovascular unit remodeling in brain disorders. Int J Mol Sci. (2014) 15:6453–74. 10.3390/ijms15046453 24743889 PMC4013640

[106] Cai W Liu H Zhao J Chen LY Chen J Lu Z. Pericytes in brain injury and repair after ischemic stroke. Transl Stroke Res. (2017) 8:107–21. 10.1007/s12975-016-0504-4 27837475 PMC5350040

[107] Yang S Jin H Zhu Y Wan Y Opoku EN Zhu L. Diverse functions and mechanisms of pericytes in ischemic stroke. Curr Neuropharmacol. (2017) 15:892–905. 10.2174/1570159X15666170112170226 28088914 PMC5652032

[108] Ottone C Krusche B Whitby A Clements M Quadrato G Pitulescu ME. Direct cell-cell contact with the vascular niche maintains quiescent neural stem cells. Nat Cell Biol. (2014) 16:1045–56. 10.1038/ncb3045 25283993 PMC4298702

[109] Azevedo PO Lousado L Paiva AE Andreotti JP Santos GSP Sena IFG. Endothelial cells maintain neural stem cells quiescent in their niche. Neuroscience. (2017) 363:62–65. 10.1016/j.neuroscience.2017.08.059 28893649 PMC6089873

[110] Zywitza V Misios A Bunatyan L Willnow TE Rajewsky N. Single-cell transcriptomics characterizes cell types in the subventricular zone and uncovers molecular defects impairing adult neurogenesis. Cell Rep. (2018) 25:2457–69 e8. 10.1016/j.celrep.2018.11.003 30485812

[111] Pinto AR Ilinykh A Ivey MJ Kuwabara JT D'Antoni ML Debuque R. Revisiting cardiac cellular composition. Circ Res. (2016) 118:400–9. 10.1161/CIRCRESAHA.115.307778 26635390 PMC4744092

[112] Hudlicka O Brown DM. Cardiac work and capillary density in normal and vascularly compromised hearts. Int J Microcirc Clin Exp. (1989) 8:365–82. 2532628

[113] Yan D Wang X Li D Liu W Li M Qu Z. Macrophages overexpressing VEGF target to infarcted myocardium and improve neovascularization and cardiac function. Int J Cardiol. (2013) 164:334–8. 10.1016/j.ijcard.2011.07.026 21794934

[114] Gladka MM Molenaar B de Ruiter H van der Elst S Tsui H Versteeg D. Single-cell sequencing of the healthy and diseased heart reveals cytoskeleton-associated protein 4 as a new modulator of fibroblasts activation. Circulation. (2018) 138:166–80. 10.1161/CIRCULATIONAHA.117.030742 29386203

[115] Skelly DA Squiers GT McLellan MA Bolisetty MT Robson P Rosenthal NA. Single-cell transcriptional profiling reveals cellular diversity and intercommunication in the mouse heart. Cell Rep. (2018) 22:600–10. 10.1016/j.celrep.2017.12.072 29346760

[116] Schepelmann M Yarova PL Lopez-Fernandez I Davies TS Brennan SC Edwards PJ. The vascular Ca2+-sensing receptor regulates blood vessel tone and blood pressure. Am J Physiol Cell Physiol. (2016) 310:C193–204. 10.1152/ajpcell.00248.2015 26538090 PMC5243220

[117] Russo D Minutolo R Clienti C De Nicola L Iodice C Savino FA. Endothelin-1 released by vascular smooth muscle cells enhances vascular responsiveness of rat mesenteric arterial bed exposed to high perfusion flow. Am J Hypertens. (1999) 12:1119–23. 10.1016/S0895-7061(99)00085-0 10604489

[118] Rudijanto A. The role of vascular smooth muscle cells on the pathogenesis of atherosclerosis. Acta Med Indones. (2007) 39:86–93. 17933075

[119] Fernandez-Hernando C Jozsef L Jenkins D Di Lorenzo A Sessa CW. Absence of Akt1 reduces vascular smooth muscle cell migration and survival and induces features of plaque vulnerability and cardiac dysfunction during atherosclerosis. Arterioscler Thromb Vasc Biol. (2009) 29:2033–40. 10.1161/ATVBAHA.109.196394 19762778 PMC2796372

[120] Kalluri AS Vellarikkal SK Edelman ER Nguyen L Subramanian A Ellinor PT. Single-cell analysis of the normal mouse aorta reveals functionally distinct endothelial cell populations. Circulation. (2019) 140:147–63. 10.1161/CIRCULATIONAHA.118.038362 31146585 PMC6693656

[121] Scott RP Quaggin ES. Review series: the cell biology of renal filtration. J Cell Biol. (2015) 209:199–210. 10.1083/jcb.201410017 25918223 PMC4411276

[122] Yang T Xu C. Physiology and pathophysiology of the intrarenal renin-angiotensin system: an update. J Am Soc Nephrol. (2017) 28:1040–9. 10.1681/ASN.2016070734 28255001 PMC5373463

[123] Dumas SJ Meta E Borri M Goveia J Rohlenova K Conchinha NV. Single-cell rna sequencing reveals renal endothelium heterogeneity and metabolic adaptation to water deprivation. J Am Soc Nephrol. (2020) 31:118–38. 10.1681/ASN.2019080832 31818909 PMC6935008

[124] Virgintino D Girolamo F Rizzi M Ahmedli N Sadowska GB Stopa EG. Ischemia/Reperfusion-induced neovascularization in the cerebral cortex of the ovine fetus. J Neuropathol Exp Neurol. (2014) 73:495–506. 10.1097/NEN.0000000000000071 24806298 PMC4122327

[125] Heng JS Rattner A Stein-O'Brien GL Winer BL Jones BW Vernon HJ. Hypoxia tolerance in the Norrin-deficient retina and the chronically hypoxic brain studied at single-cell resolution. Proc Natl Acad Sci USA. (2019) 116:9103–14. 10.1073/pnas.1821122116 30988181 PMC6500147

[126] Shiojima I Sato K Izumiya Y Schiekofer S Ito M Liao R. Disruption of coordinated cardiac hypertrophy and angiogenesis contributes to the transition to heart failure. J Clin Invest. (2005) 115:2108–18. 10.1172/JCI24682 16075055 PMC1180541

[127] Mohammed SF Hussain S Mirzoyev SA Edwards WD Maleszewski JJ Redfield MM. Coronary microvascular rarefaction and myocardial fibrosis in heart failure with preserved ejection fraction. Circulation. (2015) 131:550–9. 10.1161/CIRCULATIONAHA.114.009625 25552356 PMC4324362

[128] Hilfiker-Kleiner D Landmesser U Drexler H Molecular mechanisms in heart failure. J Am Coll Cardiol. (2006) 48:A56–66. 10.1016/j.jacc.2006.07.007

[129] Winn RK Harlan MJ. The role of endothelial cell apoptosis in inflammatory and immune diseases. J Thromb Haemost. (2005) 3:1815–24. 10.1111/j.1538-7836.2005.01378.x 16102048

[130] Lee SH Wolf PL Escudero R Deutsch R Jamieson SW Thistlethwaite AP. Early expression of angiogenesis factors in acute myocardial ischemia and infarction. N Engl J Med. (2000) 342:626–33. 10.1056/NEJM200003023420904 10699162

[131] Farbehi N Patrick R Dorison A Xaymardan M Janbandhu V Wystub-Lis K. Single-cell expression profiling reveals dynamic flux of cardiac stromal, vascular and immune cells in health and injury. eLife. (2019) 8:e43882. 10.7554/eLife.43882 30912746 PMC6459677

[132] Li Z Solomonidis EG Meloni M Taylor RS Duffin R Dobie R. Single-cell transcriptome analyses reveal novel targets modulating cardiac neovascularization by resident endothelial cells following myocardial infarction. Eur Heart J. (2019) 40:2507–20. 10.1093/eurheartj/ehz305 31162546 PMC6685329

[133] Al-Jaishi AA Oliver MJ Thomas SM Lok CE Zhang JC Garg AX. Patency rates of the arteriovenous fistula for hemodialysis: a systematic review and meta-analysis. Am J Kidney Dis. (2014) 63:464–78. 10.1053/j.ajkd.2013.08.023 24183112

[134] Protack CD Foster TR Hashimoto T Yamamoto K Lee MY Kraehling JR. Eph-B4 regulates adaptive venous remodeling to improve arteriovenous fistula patency. Sci Rep. (2017) 7:15386. 10.1038/s41598-017-13071-2 29133876 PMC5684317

[135] Zhao J Jourd'heuil FL Xue M Conti D Lopez-Soler RI Ginnan R. Dual function for mature vascular smooth muscle cells during arteriovenous fistula remodeling. J Am Heart Assoc. (2017) 6:e004891. 10.1161/JAHA.116.004891 28360226 PMC5533005

[136] McGregor H Sun Z McCoy D Kumar V Conrad M Wilson M. Endovascular biopsy and endothelial cell gene expression analysis of dialysis arteriovenous fistulas. A feasibility study. J Vasc Interv Radiol. (2018) 29:1403–9 e2. 10.1016/j.jvir.2018.04.034 30174159

[137] Crist AM Lee AR Patel NR Westhoff DE Meadows MS. Vascular deficiency of Smad4 causes arteriovenous malformations: a mouse model of hereditary hemorrhagic telangiectasia. Angiogenesis. (2018) 21:363–80. 10.1007/s10456-018-9602-0 29460088 PMC5878194

[138] Ola R Kunzel SH Zhang F Genet G Chakraborty R Pibouin-Fragner L. SMAD4 prevents flow induced arteriovenous malformations by inhibiting casein kinase 2. Circulation. (2018) 138:2379–94. 10.1161/CIRCULATIONAHA.118.033842 29976569 PMC6309254

[139] Bennett MR Sinha S Owens KG. Vascular smooth muscle cells in atherosclerosis. Circ Res. (2016) 118:692–702. 10.1161/CIRCRESAHA.115.306361 26892967 PMC4762053

[140] Misra A Feng Z Chandran RR Kabir I Rotllan N Aryal B. Integrin beta3 regulates clonality and fate of smooth muscle-derived atherosclerotic plaque cells. Nat Commun. (2018) 9:2073. 10.1038/s41467-018-04447-7 29802249 PMC5970166

[141] Tang J Wang H Huang X Li F Zhu H Li Y. Arterial Sca1(+) vascular stem cells generate *de novo* smooth muscle for artery repair and regeneration. Cell Stem Cell. (2019) 26:81–96.e4. 10.1016/j.stem.2019.11.010 31883835

[142] McDonald AI Shirali AS Aragon R Ma F Hernandez G Vaughn DA. Endothelial regeneration of large vessels is a biphasic process driven by local cells with distinct proliferative capacities. Cell Stem Cell. (2018) 23:210–25 e6. 10.1016/j.stem.2018.07.011 30075129 PMC6178982

[143] Wakabayashi T Naito H Suehiro JI Lin Y Kawaji H Iba T. CD157 marks tissue-resident endothelial stem cells with homeostatic and regenerative properties. Cell Stem Cell. (2018) 22:384–97.e6. 10.1016/j.stem.2018.01.010 29429943

[144] Hirschi KK Dejana E. Resident endothelial progenitors make themselves at home. Cell Stem Cell. (2018) 23:153–5. 10.1016/j.stem.2018.07.014 30075124

[145] Kauffenstein G Laher I Matrougui K Guérineau NC Henrion D. Emerging role of G protein-coupled receptors in microvascular myogenic tone. Cardiovasc Res. (2012) 95:223–32. 10.1093/cvr/cvs152 22637750 PMC3888209

[146] Kaur H Carvalho J Looso M Singh P Chennupati R Preussner J. Single-cell profiling reveals heterogeneity and functional patterning of GPCR expression in the vascular system. Nat Commun. (2017) 8:15700. 10.1038/ncomms15700 28621310 PMC5481776

[147] Dvorak HF. Tumor stroma, tumor blood vessels, antiangiogenesis therapy. Cancer J. (2015) 21:237–43. 10.1097/PPO.0000000000000124 26222073

[148] Hernandez SL Banerjee D Garcia A Kangsamaksin T Cheng WY Anastassiou D. Notch and VEGF pathways play distinct but complementary roles in tumor angiogenesis. Vasc Cell. (2013) 5:17. 10.1186/2045-824X-5-17 24066611 PMC3849070

[149] Caporarello N Lupo G Olivieri M Cristaldi M Cambria MT Salmeri M. Classical VEGF, Notch and Ang signalling in cancer angiogenesis, alternative approaches and future directions (Review). Mol Med Rep. (2017) 16:4393–402. 10.3892/mmr.2017.7179 28791360 PMC5646999

[150] Zhao Q Eichten A Parveen A Adler C Huang Y Wang W. Single-cell transcriptome analyses reveal endothelial cell heterogeneity in tumors and changes following antiangiogenic treatment. Cancer Res. (2018) 78:2370–82. 10.1158/0008-5472.CAN-17-2728 29449267

[151] Viallard C Larrivee B. Tumor angiogenesis and vascular normalization: alternative therapeutic targets. Angiogenesis. (2017) 20:409–26. 10.1007/s10456-017-9562-9 28660302

[152] Jonas JB Cheung CMG Panda-Jonas S. Updates on the epidemiology of age-related macular degeneration. Asia Pac J Ophthalmol. (2017) 6:493–7. 10.22608/APO.2017251 28906084

[153] Mehta S. Age-related macular degeneration. Prim Care. (2015) 42:377–91. 10.1016/j.pop.2015.05.009 26319344

[154] Enseleit F Michels S Sudano I Stahel M Zweifel S Schlager O. SAVE-AMD: Safety of VEGF inhibitors in age-related macular degeneration. Ophthalmologica. (2017) 238:205–16. 10.1159/000478665 28866675 PMC5804860

[155] Cheung GCM Lai TYY Gomi F Ruamviboonsuk P Koh A Lee KW. Anti-VEGF therapy for neovascular AMD and polypoidal choroidal vasculopathy. Asia Pac J Ophthalmol. (2017) 6:527−34. 10.22608/APO.2017260 28971633

[156] Villegas VM Aranguren LA Kovach JL Schwartz SG Flynn HW Jr. Current advances in the treatment of neovascular age-related macular degeneration. Expert Opin Drug Deliv. (2017) 14:273–82. 10.1080/17425247.2016.1213240 27434329

[157] Menon M Mohammadi S Davila-Velderrain J Goods BA Cadwell TD Xing Y. Single-cell transcriptomic atlas of the human retina identifies cell types associated with age-related macular degeneration. Nat Commun. (2019) 10:4902. 10.1038/s41467-019-12780-8 31653841 PMC6814749

[158] Sasagawa Y Danno H Takada H Ebisawa M Tanaka K Hayashi T. Quartz-Seq2: a high-throughput single-cell RNA-sequencing method that effectively uses limited sequence reads. Genome Biol. (2018) 19:29. 10.1186/s13059-018-1407-3 29523163 PMC5845169

[159] van Dijk D Sharma R Nainys J Yim K Kathail P Carr AJ. Recovering gene interactions from single-cell data using data diffusion. Cell. (2018) 174:716–29 e27. 10.1016/j.cell.2018.05.061 29961576 PMC6771278

[160] Lee JH Daugharthy ER Scheiman J Kalhor R Ferrante TC Terry R. Fluorescent in situ sequencing (FISSEQ) of RNA for gene expression profiling in intact cells and tissues. Nat Protoc. (2015) 10:442–58. 10.1038/nprot.2014.191 25675209 PMC4327781

[161] Lee JH Daugharthy ER Scheiman J Kalhor R Yang JL Ferrante TC. Highly multiplexed subcellular RNA sequencing *in situ*. Science. (2014) 343:1360–3. 10.1126/science.1250212 24578530 PMC4140943

[162] Buenrostro JD Giresi PG Zaba LC Chang HY Greenleaf JW. Transposition of native chromatin for fast and sensitive epigenomic profiling of open chromatin, DNA-binding proteins and nucleosome position. Nat Methods. (2013) 10:1213–8. 10.1038/nmeth.2688 24097267 PMC3959825

[163] Buenrostro JD Wu B Litzenburger UM Ruff D Gonzales ML Snyder MP. Single-cell chromatin accessibility reveals principles of regulatory variation. Nature. (2015) 523:486–90. 10.1038/nature14590 26083756 PMC4685948

[164] Chen X Miragaia RJ Natarajan KN Teichmann AS. A rapid and robust method for single cell chromatin accessibility profiling. Nat Commun. (2018) 9:5345. 10.1038/s41467-018-07771-0 30559361 PMC6297232

[165] Urrutia E Chen L Zhou H Jiang Y. Destin: toolkit for single-cell analysis of chromatin accessibility. Bioinformatics. (2019) 35:3818–20. 10.1093/bioinformatics/btz141 30821321 PMC6761983

[166] Bandura DR Baranov VI Ornatsky OI Antonov A Kinach R Lou X. Mass cytometry: technique for real time single cell multitarget immunoassay based on inductively coupled plasma time-of-flight mass spectrometry. Anal Chem. (2009) 81:6813–22. 10.1021/ac901049w 19601617

[167] Angelo M Bendall SC Finck R Hale MB Hitzman C Borowsky AD. Multiplexed ion beam imaging of human breast tumors. Nat Med. (2014) 20:436–42. 10.1038/nm.3488 24584119 PMC4110905

[168] Trepel M Arap W Pasqualini R. *In vivo* phage display and vascular heterogeneity: implications for targeted medicine. Curr Opin Chem Biol. (2002) 6:399–404. 10.1016/S1367-5931(02)00336-8 12023122

[169] Trepel M Arap W Pasqualini R. Exploring vascular heterogeneity for gene therapy targeting. Gene Ther. (2000) 7:2059–60. 10.1038/sj.gt.3301361 11223985

[170] Pasqualini R Moeller BJ Arap W. Leveraging molecular heterogeneity of the vascular endothelium for targeted drug delivery and imaging. Semin Thromb Hemost. (2010) 36:343–51. 10.1055/s-0030-1253456 20490984

[171] Ruoslahti E Duza T Zhang L. Vascular homing peptides with cell-penetrating properties. Curr Pharm Des. (2005) 11:3655–60. 10.2174/138161205774580787 16305501

[172] Aird WC. Endothelial cell heterogeneity. Cold Spring Harb Perspect Med. (2012) 2:a006429. 10.1101/cshperspect.a006429 22315715 PMC3253027

